# Intraperitoneal Administration of Short-Chain Fatty Acids Improves Lipid Metabolism of Long–Evans Rats in a Sex-Specific Manner

**DOI:** 10.3390/nu13030892

**Published:** 2021-03-10

**Authors:** Shrushti Shah, Tiffany Fillier, Thu Huong Pham, Raymond Thomas, Sukhinder Kaur Cheema

**Affiliations:** 1 Department of Biochemistry, Memorial University of Newfoundland, St. John’s, NL A1B 3X9, Canada; shrushti.shah@ucalgary.ca; 2 School of Science and the Environment/Boreal Ecosystem Research Initiative, Grenfell Campus, Memorial University of Newfoundland, Corner Brook, NL A2H 5G4, Canada; tfillier@grenfell.mun.ca (T.F.); tpham@grenfell.mun.ca (T.H.P.); rthomas@grenfell.mun.ca (R.T.)

**Keywords:** gene regulation, gut microbial metabolites, lipids, sex-specific effects, short-chain fatty acids

## Abstract

Short-chain fatty acids (SCFAs) are microbial metabolites, mainly generated by the action of gut microbiota on dietary fibers. Acetate, propionate, and butyrate are the three main SCFAs produced typically in a 60:20:20 molar ratio in the colon. Acetate, propionate, and butyrate, when given individually as supplements, have shown a protective role in obesity and hyperglycemia; however, the sex-specific effects of a mixture of SCFAs, when given in 60:20:20 ratio, on the regulation of lipid metabolism and lipid profile are not known. Male and female Long–Evans rats were given a mixture of SCFAs (acetate, propionate, and butyrate; molar ratio 60:20:20) each day for seven days intraperitoneally; plasma and hepatic lipids, gene expression, and lipidomics profile were analyzed. SCFAs significantly decreased plasma and hepatic triglycerides and cholesterol in males, whereas the fatty acyl composition of cholesteryl esters, triglycerides, and phospholipids was modulated in females. SCFAs decreased the mRNA expression of hepatic acetyl-CoA carboxylase-1 in both males and females. Our findings demonstrate for the first time that SCFAs (60:20:20) improved plasma and hepatic lipid levels and fatty acyl composition in a manner that may provide cardio-protective and anti-inflammatory effects in both sexes, via independent mechanisms.

## 1. Introduction

Gut-microbiota and their metabolites have recently gained much interest in the scientific community due to their roles in the regulation of lipids and glucose metabolism [1,2]. The digestion of dietary fibers and other complex carbohydrates by gut microbiota produces short-chain fatty acids (SCFAs) as one of its many metabolites. Acetate, propionate, and butyrate are considered as the three main SCFAs generated in varying abundance in the colon, with the highest concentrations in the proximal region (70 to 140 mM), followed by the distal region (20 to 70 mM) [3]. Once produced in the gut, acetate and propionate are mainly taken up by the liver from portal circulation, where these act as a substrate for de novo synthesis of longer-chain fatty acids (LCFAs), and hepatic gluconeogenesis, respectively [4,5]. On the other hand, most of the butyrate produced by gut microbiota is taken up by colonocytes for energy [6].

Although, much of the biochemistry of SCFAs is known, accurate production rates of acetate, propionate, and butyrate in humans are not conclusive [7]. This is because of the inadequate data from human intestinal samples and also the variability of the microbiome in healthy individuals [5]. Rodent [8] and swine [9] models have indicated that the molar ratio of acetate:propionate:butyrate produced in the colon is approximately 60:20:20; human subjects fed various types of dietary fibers have supported these results upon analyses of their blood and fecal samples [6,10]. However, diet, microbiome, and other environmental factors are suggested to induce gut dysbiosis, thereby altering the abundance/ratio of SCFAs [5], which is associated with various metabolic and inflammatory diseases such as obesity, type 2 diabetes (T2D), inflammatory bowel disease, and other gastrointestinal disorders [5,11]. This suggests that the positive health benefits of SCFAs, such as prevention of body-weight gain, improved lipid oxidation, and their anti-inflammatory role in autoimmune diseases, may be associated with both the type of microbiota and the generation of SCFAs in the 60:20:20 molar ratio [5,12].

SCFAs act as substrates for glucose, cholesterol, and lipid synthesis [13], thereby regulating several metabolic pathways, including but not limited to, hepatic lipogenesis, adipogenesis, and lipolysis in the adipose tissue [4,14]. Acetate has been shown to decrease serum cholesterol levels in rats by downregulating 3-hydroxy-3-methylglutaryl-CoA reductase (Hmgcr) enzyme—a rate-limiting enzyme involved in de novo cholesterol synthesis [15]. Acetate or propionate, given orally, reduced glycemia in diabetic hyperglycemic mice [16]. Acetate and propionate have also been shown to reduce plasma free fatty acids by activating fatty acid oxidation and inhibiting lipolysis in adipose tissue in a human white adipocyte model [14]. Individual supplementation of all three SCFAs (acetate, propionate, and butyrate; 5% *w*/*w*) and their admixture (3:1:1) in high-fat diet (HFD) caused a shift from lipogenesis to fatty acid oxidation and prevented HFD-induced obesity and insulin resistance in mice [4]. Furthermore, epithelial cells in rat colon showed significantly higher incorporation of acetate and butyrate into phospholipids compared to free fatty acids and triacylglycerols (TG), indicating a role of SCFAs in membrane synthesis [17]. These studies have clearly shown that individual SCFAs regulate metabolic pathways; however, SCFAs (acetate, propionate, and butyrate) are generated as a mixture, generally in the molar ratio of 60:20:20, but there is no data to support the regulation of metabolic pathways by a mixture of SCFAs in this molar ratio. Moreover, most of the pre-clinical and clinical studies of SCFAs on the regulation of lipid metabolism have focused on males [15,18]. Sex hormones are well documented to regulate metabolic pathways, and females have a higher sensitivity toward insulin compared to males [19]. In this study, we investigated the sex-specific effects of a mixture of SCFAs on the metabolic regulation and lipid profile of Long–Evans rats. We hypothesized that acetate, propionate, and butyrate, when administered in a molar ratio of 60:20:20, will decrease plasma and hepatic cholesterol and TG levels, and plasma glucose levels, in both male and female Long–Evans rats. Since SCFAs are used for fatty acid synthesis [20], we further hypothesized that SCFAs will alter plasma and hepatic lipid profiles, which may be important in metabolic regulation of the host [21].

## 2. Materials and Methods

### 2.1. Animals and Experiment Design

Long–Evans rats (12 males and 12 females; 45–50 days old) were obtained from Charles River Laboratories (Quebec, QC, Canada) and were housed in individual cages in a well-ventilated room and maintained under standard laboratory conditions (21 °C; 12 h light/dark cycle). After one week of acclimatization, male and female rats were divided into control and treatment groups (*n* = 6 in each group). Control rats received 0.1M phosphate-buffered saline (PBS; pH 7.4) solution at 2 mg/kg body mass, whereas treatment groups received a mixture of sodium acetate (Cat #S2889, Sigma-Aldrich, Oakville, ON, Canada), sodium propionate (Cat #P1880, Sigma-Aldrich, Oakville, ON, Canada), and sodium butyrate (Cat #B6887, Sigma-Aldrich, Oakville, ON, Canada), dissolved in 0.1 M PBS in the molar ratio of 60:20:20, respectively. Treatment was given at a dose of 500 mg/kg body mass, as per previous publications [22]. Intraperitoneal (I.P.) administration of treatment was carried out each day for seven days at 7:00 a.m. Rats were weighed each morning before administration by I.P. injection, and the amount of vehicle control and treatment was calculated according to the body weight. Food intake was measured daily. All the animal procedures were in accordance with the Guidelines of the Canadian Council on Animal Care and were approved by the Animal Care Committee of Memorial University of Newfoundland (protocol #16-01-RT). Animals had free access to chow diet (Cat #7012; Teklad Laboratory Animal Diets, Madison, WI, USA) and water throughout the experimental period.

### 2.2. Blood and Tissue Sampling

After one week of treatment, rats were killed by cardiac puncture under isoflurane in a non-fasted state. Blood samples were collected into tubes containing EDTA (0.1%, pH 7.4) and centrifuged for 10 min at 2100× *g* (4 °C) to separate plasma. Liver and abdominal fat was collected, weighed, snap-frozen in liquid nitrogen, and stored at −80 °C until further analysis.

### 2.3. Analysis of Biochemical Parameters

Total lipids were extracted from liver and adipose tissue using the Folch method [23] as per our previous publications [24]. Plasma and hepatic total cholesterol (TC) and TG, and plasma non-esterified fatty acids (NEFAs) and glucose were assayed according to the manufacturers’ instructions, using commercially available kits: TC kit #234-60 (Sekisui Diagnostics, Lexington, MA, USA); TG kit #236-17 (Sekisui Diagnostics, Lexington, MA, USA); NEFA kit #278-76491 (WAKO chemicals, Richmond, VA, USA); and glucose kit #10009582 (Cayman chemicals, Ann Arbor, MI, USA). Free cholesterol (FCh) and cholesteryl esters (ChE) were quantified in plasma and liver samples using thin-layer chromatography coupled to a flame ionization detector (TLC-FID) [25]. A standard mixture containing 1 mg/mL of cholesterol, TG, ChE, free fatty acids, monoacylglycerols, and diacylglycerols was used. Conditions and procedure for TLC-FID were followed as per Marshall et al. [26]. Analysis of peak areas was done in Iatroscan MK-6s using Peak 432-64 bit software.

### 2.4. Gene Expression Analysis

Total RNA was extracted from liver and adipose tissue samples using the Trizol method [27]. The integrity and concentration of the extracted RNA were assessed using gel electrophoresis (1.2% agarose gel) and NanoDrop 2000 (Thermo Scientific, St Peters, MO, USA), respectively. Genomic DNA contamination was removed by DNase enzyme treatment (Cat #M610A 12358503; Promega, Madison, WI, USA). Reverse transcription was performed to generate cDNA (Cat #A3500; Promega, Madison, WI, USA), which was later amplified for quantitative real-time PCR (qPCR) using iQ-SYBR Green Supermix (Cat #170-8880; Bio-Rad Laboratories. Inc., Hercules, CA, USA) as per our previous publications [28]. Primers for lipogenic and adipogenic genes (Supplementary Table S1) were designed using National Center for Biotechnology Information primer blast (www.ncbi.nlm.gov/tools/primerblast/, accessed on 4 February 2021) and obtained from IDT technologies (Coralville, IA, USA). Relative mRNA expressions for hepatic genes were standardized to *β-actin* as the housekeeping gene, whereas glyceraldehyde-3-phosphate dehydrogenase (*Gapdh*) was used as a housekeeping gene for adipose tissue. The ∆∆C_T_ method [29] was used to calculate relative gene expression.

### 2.5. Lipidomics Analysis

HPLC-grade acetic acid, formic acid, ammonium acetate, ammonium formate, acetonitrile, chloroform, and methanol were purchased from Fisher Scientific, ON, Canada. Deionized water was obtained from a PURELAB Purification System (ELGA Lab-water, ON, Canada) for solution preparation. Each lipid class’ standards were purchased from Avanti Polar Lipids (Alabaster, AL, USA). Plasma and hepatic lipids were extracted using the Bligh and Dyer method [30]. A mixture of internal and external standard was prepared as per our previous publications [31]. The complex lipid standard mixture and lipids extracted from the samples were analyzed via C30 reverse-phase (C30 RP) chromatography, coupled with ultra-high-performance liquid chromatography and heated electrospray ionization high-resolution accurate mass tandem mass spectrometry (UHPLC-C30RP-HESI-HRAM-MS/MS). A Q-Exactive Orbitrap mass spectrometer (Thermo Scientific, MO, USA) coupled with an automated Dionex UltiMate 3000 UHPLC system controlled by Chromeleon software was used for the lipid analysis [32]. The parameters listed in Supplementary Table S2 were used for the Orbitrap mass spectrometer. XCalibur 4 (ThermoScientific, MO, USA) and LipidSearch 4.1 (Thermo-Scientific, Mississauga, ON, Canada) software were used to acquire and process all MS data. The identification and semi-quantification of all the lipid classes and their sub-classes, along with lipid molecular species within each class were determined using LipidSearch software and manual confirmation was done using XCalibur 4. Phosphatidycholine (PC), lyso PC (LPC), TG, and ChE lipid classes were identified in positive ion mode as hydrogen ([M + H]^+^) and ammonia ([M + NH_4_]^+^)adducts, whereas phosphatidylethanolamine (PE), lyso PE (LPE), phosphatidylserine, phosphatidylinositol, and phosphatidic acids classes were identified in negative ion mode ([M − H]^−^, [M + HCOO]^−^, and [M − 2H]^2−^). Following identifications, the observed lipid classes and molecular species were aligned and merged according to our previous publication [31]. The fatty acids present in the molecular species found in each lipid class of the samples evaluated were identified based on the fragmentation patterns of the MS/MS spectra and manually confirmed using XCalibur 4.

### 2.6. Data Analysis

Statistical analyses were performed using two-way analysis of variance (ANOVA) (treatment and sex as the two parameters) followed by Tukey’s post hoc analysis using GraphPad Prism (Version 6.0; GraphPad Software Inc., La Jolla, CA, USA). The significance level was set at *p* < 0.05; different superscripts indicate significant differences amongst groups. The Grubbs’ outlier test was performed for all lipidomics data using XLSTAT software (Premium version, Addinsoft, Paris, France). Principal component analysis (PCA) was used to group the lipid outputs based on similarities or differences, where more than 60% of total variance in the data was used as a criterion for ordination of the lipidomics data during principal component analysis (PCA). Two-way ANOVA was used to assess the significance of the clusters or groups observed on selected lipid molecular species or classes, following PCA analysis. Lipid molecular species clustered around control and treatment quadrants in the PCA biplot were selected for post hoc analysis.

## 3. Results

### 3.1. Effect of SCFAs on Body Weight, Food Intake, Glucose, and NEFA Levels

SCFAs administration had no significant effect on body weight or food intake (Table 1) at the end of the study period (day 7), in both males and females, compared to their respective control groups. However, females had a significantly lower body weight and food intake, compared to their male counterparts. SCFAs had no significant effect on liver or abdominal fat weight (relative to body weight) in either males or females (Table 1). SCFA-treated males showed significantly lower (*p* < 0.05) levels of plasma glucose, compared to their control group. However, there was no effect of SCFAs on the plasma glucose levels of females, compared to their control group (Table 1). There was no effect of SCFAs on NEFA in both males and females, compared to their respective control groups (Table 1).

### 3.2. SCFAs Had Sex-Specific Effects on Cholesterol Metabolism

**Figure 1:**
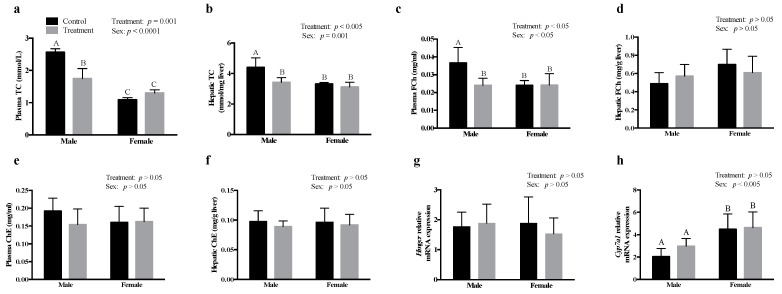
Intraperitoneal administration of SCFAs had sex-specific effects on cholesterol metabolism. Effect of SCFAs on, (**a**) plasma total cholesterol (TC), (**b**) plasma FCh, (**c**) plasma cholesteryl esters (ChE), (**d**) hepatic TC, (**e**) hepatic FCh, (**f**) hepatic ChE, and the relative mRNA abundance of (**g**) *Hmgcr* and (**h**) *Cyp7a1*. The mRNA expression data are presented relative to β-actin. Data were analyzed using two-way ANOVA followed by Tukey’s post hoc test. All data are expressed as mean ± SD. Different superscripts indicate significant differences amongst groups; *p* < 0.05 was considered significant (*n* = 6). *Cyp7a1—*cholesterol 7 α-hydroxylase, FCh—free cholesterol, *Hmgcr—*3-hydroxy-3-methylglutaryl-CoA reductase, SCFAs—short-chain fatty acids.

Males treated with SCFAs revealed a significant decrease in plasma (*p* = 0.001) and hepatic (*p* < 0.005) TC concentrations, compared to their respective controls; however, SCFA-treated females revealed no change in plasma and hepatic TC concentrations, compared to their respective controls (Figure 1a,b, respectively). Similar to the effects of SCFAs on plasma TC in males, the plasma FC levels were also decreased by SCFAs in males, compared to that in the control group (*p* < 0.05), while there was no effect of SCFAs on plasma FCh in females (Figure 1c). There was no effect of SCFAs on hepatic FCh (Figure 1d) in males or females. Furthermore, there was no effect of SCFAs on plasma (Figure 1e) or hepatic ChE (Figure 1f) in both males and females, compared to their respective controls. SCFA treatment had no effect on the mRNA expression of *Hmgcr* and cholesterol 7 α-hydroxylase (*Cyp7a1*) in both males and females (Figure 1g,h, respectively). We also observed that females had higher mRNA expression of *Cyp7a1*, compared to both male control and male treatment groups.

### 3.3. SCFAs Altered Plasma and Hepatic ChE Fatty Acid Composition

**Figure 2:**
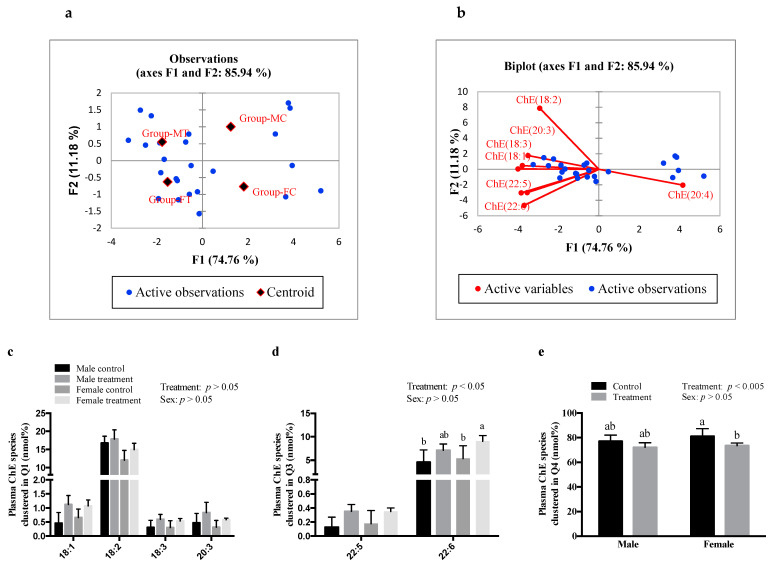
Intraperitoneal administration of SCFAs had a significant effect on plasma ChE fatty acyl composition. Principal component analysis showing (**a**) observation plot and (**b**) biplot of TG molecular species clustered in: (**c**) Q1—quadrant 1, (**d**) Q2—quadrant 2, and (**e**) Q4—quadrant 4. Data were analyzed using multivariant analysis, and two-way ANOVA followed by Tukey’s post hoc test. All data are expressed in mean ± SD. Different superscripts indicate significant difference amongst groups; *p* < 0.05 was considered significant (*n* = 6). ChE—cholesteryl esters, FC—female control, FT—female treatment, MC—male control, MT—male treatment.

SCFAs had sex-specific effects on plasma and hepatic ChE fatty acid composition. The observation chart and biplot obtained by PCA for plasma ChE molecular species is shown in Figure 2a,b, respectively. Each of the four groups (male control—MC, male treatment—MT, female control—FC, female treatment—FT) clustered in different quadrants of both the observation and biplots indicating sex differences in response to SCFAs treatments. Post hoc analysis showed that SCFA treatment had higher abundance of plasma ChE composed of 18:1, 18:2, 18:3, and 20:3 species, compared to respective controls; the values however were not significantly different (Figure 2c). Males treated with SCFAs showed no significant effect on the levels of 20:4 or 22:6 ChE in plasma (Figure 2d,e, respectively). However, females showed significantly higher relative abundance of 22:6 ChE (*p* < 0.05; Figure 2d), and lower abundance of 20:4 ChE (*p* < 0.005; Figure 2e) in plasma, compared to the control group. There was no effect of SCFAs on the hepatic ChE composed of 18:1, 18:2, 18:3, 20:4, 22:5, or 22:6 in both males and females (Supplementary Figure S1).

### 3.4. SCFAs Had Sex-Specific Effects on TG Metabolism

**Figure 3:**
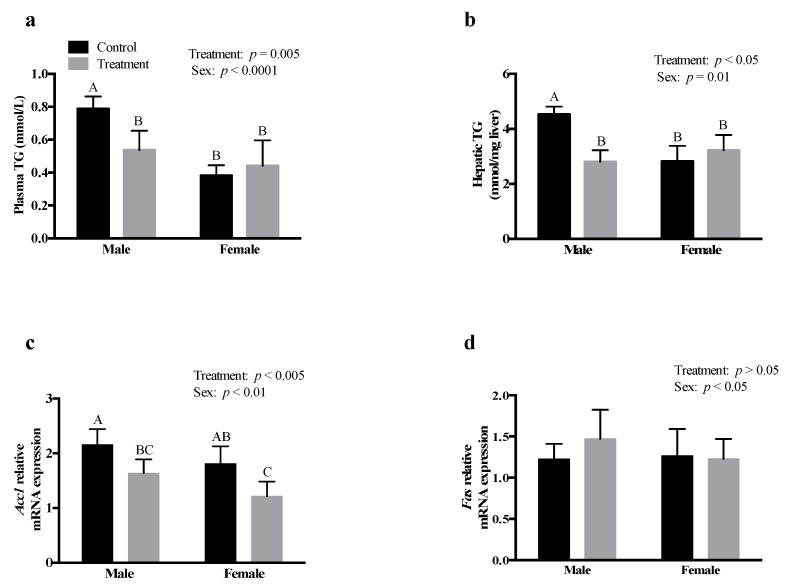
Intraperitoneal administration of an SCFA mixture had sex-specific effects on TG metabolism. Effect of SCFAs on, (**a**) plasma TG, (**b**) hepatic TG, and the relative mRNA expression of (**c**) *Acc1*, and (**d**) *Fas*. Data were analyzed using two-way ANOVA followed by Tukey’s post hoc test. The mRNA expression data are presented relative to β-actin. All data are expressed as mean ± SD. Different superscripts indicate significant difference among groups; *p* < 0.05 was considered significant (*n* = 6). *Acc1—*acetyl-CoA carboxylase subform1, *Fas*—fatty acid synthase, SCFAs—short-chain fatty acids, TG—triglycerides.

SCFA treatment significantly decreased plasma (*p* = 0.005) and hepatic (*p* < 0.05) TG concentrations in males; however, there was no effect on plasma and hepatic TG concentrations in females, compared to their respective control groups (Figure 3a,b, respectively). SCFA treatment significantly decreased the mRNA expression of hepatic acetyl-CoA carboxylase-1 (*Acc1*) in both males and females, compared to their respective controls (*p* < 0.005; Figure 3c).

However, there was no effect of SCFA treatment on the hepatic fatty acid synthase (*Fas*) mRNA expression in both males and females (Figure 3d). There was no effect of SCFA treatment on adipose tissue TG and the mRNA expression of peroxisome proliferator-activated receptor-γ (*Pparg*) (Supplementary Figure S2) in both males or females, compared to their respective controls.

### 3.5. SCFAs Had Sex-Specific Effects on TG Fatty Acid Composition

**Figure 4:**
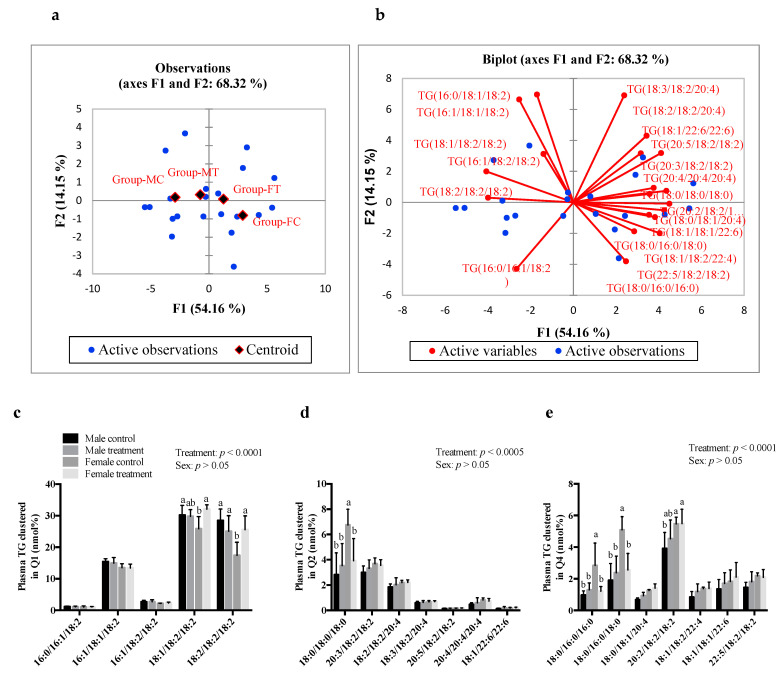
Intraperitoneal administration of SCFAs had a significant effect on plasma TG fatty acyl composition in females. Principal component analysis showing (**a**) observation plot and (**b**) biplot of TG molecular species clustered in: (**c**) Q1—quadrant 1, (**d**) Q2—quadrant 2, and (**e**) Q4—quadrant 4. Data were analyzed using multivariant analysis and two-way ANOVA followed by Tukey’s post hoc test. All data are expressed as mean ± SD. Different superscripts indicate significant difference amongst groups; *p* < 0.05 was considered significant (*n* = 6). FC—female control, FT—female treatment, MC—male control, MT—male treatment, SCFAs—short-chain fatty acids, TG—triglycerides.

The PCA output of TG molecular species suggested that there was no significant effect of SCFAs on males (MC and MT groups were found in quadrant 1). However, FT and FC groups were found in quadrants 2 and 4 respectively, indicating an effect of SCFAs on females (Figure 4a,b, respectively). Post hoc analysis showed that there was no effect of SCFA treatment on plasma TG composed of saturated fatty acids (SFAs) and polyunsaturated fatty acids (PUFAs) in males, compared to its controls. However, SCFA-treated females showed a significantly higher abundance of TG species composed of 18:1/18:2/18:2 and 18:2/18:2/18:2 (*p* < 0.05; Figure 4c), while the relative abundance of species composed of SFAs at all three *sn*-positions (TG—18:0/18:0/18:0, −18:0/16:0/16:0, and −18:0/16:0/18:0) was significantly lower (Figure 4d,e, respectively). There was no difference in the relative abundance of hepatic TG species composed of SFAs, monounsaturated fatty acids (MUFAs), and PUFAs (Supplementary Figure S3).

### 3.6. SCFAs Had Sex-Specific Effects on Plasma and Hepatic PC Fatty Acid Composition

**Figure 5:**
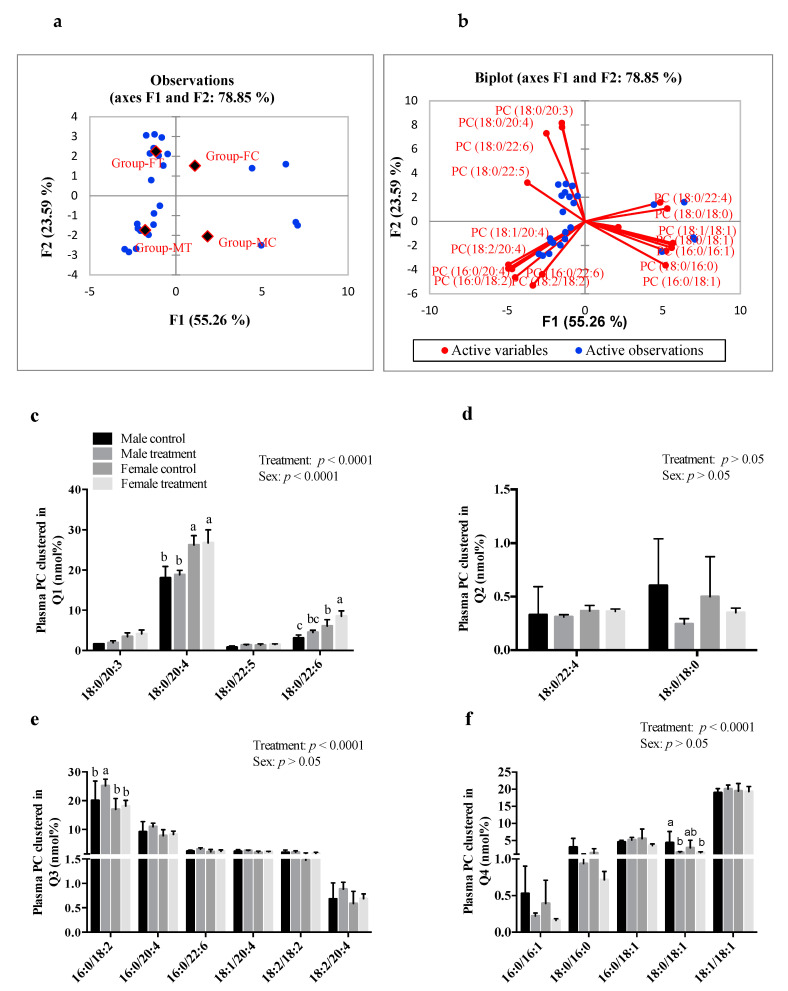
Intraperitoneal administration of SCFAs showed a significant effect on plasma phosphatidylcholine (PC) molecular species in both males and females. Principal component analysis showing (**a**) observation chart and (**b**) biplot of PC species clustered in: (**c**) Q1—quadrant 1, (**d**) Q2—quadrant 2, (**e**) Q3—quadrant 3, and (**f**) Q4—quadrant 4. Data were analyzed using multivariate analysis and two-way ANOVA followed by Tukey’s post hoc test. All data are expressed as mean ± SD. Different superscripts indicate significant difference amongst groups; *p* < 0.05 was considered significant (*n* = 6). SCFAs—short-chain fatty acids.

The observation chart and biplot obtained by PCA for plasma PC molecular species are shown in Figure 5. Each of the four groups were clustered in different quadrants, indicating that SCFA treatments altered plasma PC in both males and females (Figure 5a). Males showed no effect of SCFAs on the relative abundance of PC species composed of n-3 PUFAs (−20:3, −22:5, or −22:6) at *sn*-2 position and 18:0 acyl chain in *sn*-1 position (clustered in quadrant 1), while females revealed a significant increase (*p* < 0.0001) in the abundance of 18:0/22:6 PC species after SCFAs treatment, compared to their respective control groups (Figure 5c). Males treated with SCFAs showed a significant increase (*p* < 0.0001) in the abundance of plasma 16:0/18:2 PC (Figure 5e) relative to 18:0/18:1 PC, which showed a decrease in abundance (*p* < 0.0001), compared to its controls (Figure 5f). There was no change in the abundance of PC species composed of *n-6* PUFAs (16:0/20:4, 18:1/20:4, 18:1/20:4, 18:2/18:2, 18:2/20:4) found to cluster in quadrant 3 (Figure 5e), or those species composed of MUFAs (16:0/16:1, 16:0/18:1, 18:1/18:1) and clustered in quadrant 4 of the PCA biplot (Figure 5f) in males or females. There was no effect of SCFA treatment on the relative abundance of hepatic PC, or plasma and hepatic LPC, n-6 PUFAs (18:2, 20:3, and 20:4) or *n-3* PUFAs (22:5 and 22:6) in either males or females; compared to their respective controls (Supplementary Figures S4 and S5).

## 4. Discussion

Symbiosis amongst microbial communities responsible for generating SCFAs is a key factor to generate acetate, propionate, and butyrate in the molar ratio of 60:20:20 [2,5]. In humans, potential health benefits associated with SCFAs such as prevention of body-weight gain and improving insulin sensitivity have been linked to the presence of SCFAs in a molar ratio of 60:20:20 [2,33]. However, majority of the studies to investigate the mechanisms of action of SCFAs in metabolic regulation have focused on individual actions of acetate, propionate, or butyrate [4,15,18]. We investigated the effects of a mixture of SCFAs in the molar ratio of 60:20:20 on metabolic regulation, and the mechanisms of action using Long–Evans rats.

We found that a mixture of SCFAs had no effect on food intake and body weight of both males and females. Others have reported that sodium butyrate, when given in an HFD at 5% (*w*/*w*), had no significant effect on body weight and food intake [34]. Similarly, acetate, propionate, or butyrate, when given individually to C57BL/6 mice (sex not mentioned) had no effect on body weight and food intake, compared to mice fed only an HFD [4]. However, it has been reported that when acetate was administered I.P. (500 mg/kg body weight), there was an increase in plasma acetate levels in male C57BL/6 mice, with a significant reduction in acute food intake at one and two hours post injection [22]. These authors suggested that an increase in plasma acetate levels acted as an anorexic signal in the gut–brain axis, thereby reducing food intake. However, we did not find an increase in plasma acetate levels, or in the other SCFAs (data not shown), which may explain no effect on food intake or body-weight gain. SCFAs have also been shown to stimulate fat oxidation in the liver and adipose tissue, along with a decrease in adipose tissue *Pparg* mRNA expression and activity [4]. *Pparg* has an important role in the process of adipogenesis and also glucose homeostasis; deletion of adipose tissue *Pparg* decreased weight gain and insulin resistance in mice and, thus, protected against diet-induced obesity [35]. We observed no change in adipose tissue *Pparg* mRNA expression or adipose TG concentration in our study, which may further explain no change in body weight after SCFA treatment.

SCFAs significantly reduced plasma glucose levels in males, while this effect was not found in females. Previous studies have suggested that the effects of SCFAs on glucose metabolism are dependent on the ratio of propionate to butyrate and not on propionate or butyrate alone [33]. Similarly, I.P. administered acetate alone had no effect on blood glucose concentrations of male C57BL/6 mice [22]. It is evident from our study that a combination of SCFAs is required to influence blood glucose concentrations. We observed sex-specific effects of SCFAs on glucose concentrations, where females showed no effect. It is well-established that females have higher whole-body insulin sensitivity and glucose effectiveness, compared to males [19]. Thus, sex-specific differences in plasma glucose levels could be due to the role of estrogen in maintaining glucose homeostasis in females.

As observed for plasma glucose levels, SCFAs also significantly decreased plasma and hepatic TC levels in males, while there was no effect in females. SCFAs, especially acetate, has previously been shown to reduce plasma cholesterol levels, which was not due to a decrease in cholesterol synthesis or an increase in *Cyp7a1* mRNA expression [15] We also found no effect of SCFAs on the mRNA expression of *Hmgcr* or *Cyp7a1*. Others, however, have reported that a dietary acetate- or propionate-mediated decrease in serum cholesterol levels is due to a decrease in the enzyme activity of *Hmgcr* and 3-hydroxy-3-methylglutaryl-CoA synthase [5]. *Hmgcr* is regulated at both the transcriptional and posttranscriptional levels [36]. We only measured the mRNA expression of *Hmgcr*; however, it is possible that the regulation is at the Hmgcr enzyme activity level. In the study by Fushimi et al. [15], acetate was also found to increase fecal bile content; however, they did not find any change in *Cyp7a1* activity or mRNA levels. We also observed that there was higher mRNA expression of *Cyp7a1* in females compared to that in males. Our results are in line with other animal studies showing that female mice have a larger total bile acid pool, but females catabolize less cholesterol via bile acid production compared to males [37]. Individual supplementation of acetate, propionate, or butyrate has been shown to decrease plasma TC, with a concomitant increase in fecal bile acids [18], and the decrease in plasma TC levels was due to the upregulation of sterol regulatory element-binding protein-2, low-density lipoprotein receptor, and *Cyp7a1* mRNA expression. Although we did not measure fecal bile acids, it is apparent that SCFAs, whether given alone or in combination, reduce cholesterol levels.

We also found a decrease in plasma FCh levels in males after treatment with SCFAs, which may be due to an increase in lecithin-cholesterol acyltransferase (*Lcat*) activity. The cholesterol esterification reaction is catalyzed via Lcat and acyl-CoA acyltransferase enzymes in plasma and liver, respectively [38]. *Lcat* is involved in the hydrolysis of fatty acid at *sn*-2 position of phospholipids, which then transfers the hydrolyzed fatty acid to cholesterol to yield ChE [39]. ChE is the storage form of cholesterol, and excess accumulation of cholesterol is associated with a higher risk of heart diseases [40]. We did not find any change in plasma and hepatic levels of ChE, suggesting that the decrease in FCh might be responsible for an overall decrease in TC levels in males treated with SCFAs. Interestingly, we did not observe an effect of SCFAs on plasma and hepatic TC levels in females. Higher levels of estrogen in females are responsible for reduced hepatic lipogenesis and increased lipolysis in adipocytes, which leads to a decreased de novo synthesis of lipids [41].

Similar to our finding with glucose and cholesterol levels, SCFAs reduced plasma TG levels in males, with no effect in females, compared to their respective controls. Previous studies have suggested that the effect of SCFAs on TG metabolism is either due to increased fatty acid oxidation in multiple tissues or due to decreased fat storage in adipose tissues [4,5]. We did not observe an effect of SCFAs on adipose tissue TG levels or on the mRNA expression of *Pparg* in both males and females. This discrepancy may be because the majority of the studies on SCFAs treatment used animal models that were fed an HFD, while animals in the current study were fed a standard chow diet. Moreover, majority of the studies investigated the effects of individual SCFAs. Interestingly, we found that SCFAs reduced the mRNA expression of hepatic *Acc1* in both males and females. Our findings suggest that the decrease in plasma and hepatic TG levels is more likely due to the inhibition of *Acc1*, the rate limiting enzyme in fatty acid synthesis [42]. Our results further confirm that SCFAs have sex-specific effects on TG metabolism, consistent with the effects on glucose and cholesterol metabolism. Males tend to oxidize more lipids than females, which may be responsible for different effects of SCFAs on lipid metabolism in males vs. females [19,43]. Acetate gets incorporated into LCFAs in the liver and is then utilized for the formation of complex lipids during the process of de novo lipogenesis and cholesterol synthesis [13], while propionate and butyrate inhibit these processes. It has been suggested that a combination of SCFAs may negate the effects of individual SCFAs on lipid metabolism [33]. In the current study, a combination of acetate, propionate, and butyrate was used, which may explain the differences in the effects observed on cholesterol and TG metabolism in liver and adipose tissue, compared to previously published studies that evaluated the effects of individual SCFAs on lipid metabolism [15,17].

Since acetate is incorporated into LCFAs, which are then used to form complex lipids [5], we investigated the effects of SCFAs on the plasma and hepatic lipidomics profile. We found that SCFAs reduced the incorporation of inflammatory 20:4 fatty acids in ChE, while increasing the incorporation of 22:6 fatty acids in ChE in females. The fatty acid composition of plasma and hepatic ChE is associated with insulin resistance and blood glucose levels [25]. A clinical trial conducted in Finland found that Finnish diabetic subjects had higher levels of serum ChE containing 18:3, 20:3, and 20:4 fatty acids, compared to non-diabetic subjects [44]. It is known that 20:4 ChE acts as a substrate for oxidizing agents, such as 12- or 15-lipoxygenase; thus, the metabolites generated by 20:4 ChE are associated with atherosclerosis and modulation of the anti-inflammatory response cascade [45]. On the other hand, *n-3* PUFAs, such as 20:5 and 22:6, lower the propensity for inflammatory metabolites [46]. Butyrate, when administered in db/db diabetic mice with obesity via the I.P. route (1 g/kg), reduced the mRNA expression of inflammatory cytokines (interleukin 1, interleukin 6, and tumor necrosis factor α) in adipose tissue [47]. Thus, it is likely that SCFAs inhibit the inflammatory cascade, thereby eliciting beneficial health effects.

Consistent with ChE, we also found that SCFA treatment caused a significant increase in plasma PC composed of *n-3* PUFA, with a significant increase in 16:0/22:6, 18:0/22:5, and 18:0/22:6 PC. It is well known that *n-3* PUFAs possess anti-inflammatory effects and protect against heart disease [48]. A recent study designed to investigate early onset marker for the side-effects of tamoxifen (a breast cancer drug) found that plasma PC species containing 20:4 fatty acids was reduced in Sprague–Dawley rats [49], suggesting that alterations in PC fatty acyl composition is of significance. The increase in 16:0/18:2, 16:0/22:6, 18:0/22:5, and 18:0/22:6 PC could be due to the effect of SCFAs on the regulation of elongation and desaturation enzymes. Chalil et al. [50] reported that an increase in plasma 16:0/22:6 PC levels in pregnant rats was due to increased hepatic phosphatidylethanolamine N-methyltransferase (*Pemt*) expression and *Pemt* activity, along with increased fatty acid desaturase protein levels. It would be interesting to look into hepatic *Pemt* and other enzymes involved in PUFA synthesis in the future because our results show that SCFAs increased incorporation of n-3 PUFAs irrespective of 16:0 or 18:0 at the sn_1_ position of the glycerol moiety in PC. In addition to the involvement of PC species in inflammatory processes, these phospholipids have also been suggested to have beneficial effects in the therapy of Alzheimer’s and schizophrenia-like neurodegenerative diseases that are characterized by impaired synthesis of PC and LPC [51,52]. We found that along with increasing relative abundance of plasma 18:0/22:6 PC, SCFAs also increased the abundance of hepatic 20:4 LPC in females. Previous studies on mice deficient in lysophosphatidylcholine acyltransferase-3, an enzyme responsible for the re-esterification of LPC to PC showed reduced levels of plasma lipids (cholesterol, TG, and phospholipids) [53] and also decreased the production of phospholipids containing 20:4 fatty acids [54]. Thus, LPCs are associated with the secretion of enzymes involved with the absorption and transport of lipids and in the delivery of PUFAs such as DHA to the brain [42]. It is thus possible that an SCFA mixture in the 60:20:20 molar ratio enhances the transport of PC and LPC species in circulation, and possibly to the brain.

Even though SCFA-treated females showed no change in plasma and hepatic TG levels, the relative abundance of TG species composed of 16:0 and 18:0 was significantly decreased after SCFAs treatment, suggesting that SCFAs might have an effect on the packaging and secretion of lipoproteins (such as VLDL). This is because lipoproteins help the transport of TG and other lipids into the circulation and other peripheral tissues [55]. Given that lipids packaged for release in the blood stream and distribution to peripheral tissues are acted upon by lipases (lipoprotein lipase, hepatic lipase), it is likely that SCFAs impact lipases. We observed no significant differences in the relative abundance of hepatic TG species composed of SFAs, MUFAs, *n-6* PUFAs, or *n-3* PUFAs. This indicates that SCFAs did not serve as a substrate for TG synthesis or, alternately, for the esterification of fatty acids. The endogenous synthesis of TG occurs mainly in the liver, adipose tissue, and intestine [56]. In this study, the route of administration being I.P., we expected changes in the fatty acid synthesis pathway in liver. We found that SCFA treatment showed a significant decrease in hepatic *Acc1* mRNA expression in both males and females; however, only males displayed a decrease in hepatic TG levels, while females showed a decrease in TG species containing SFAs.

**Figure 6:**
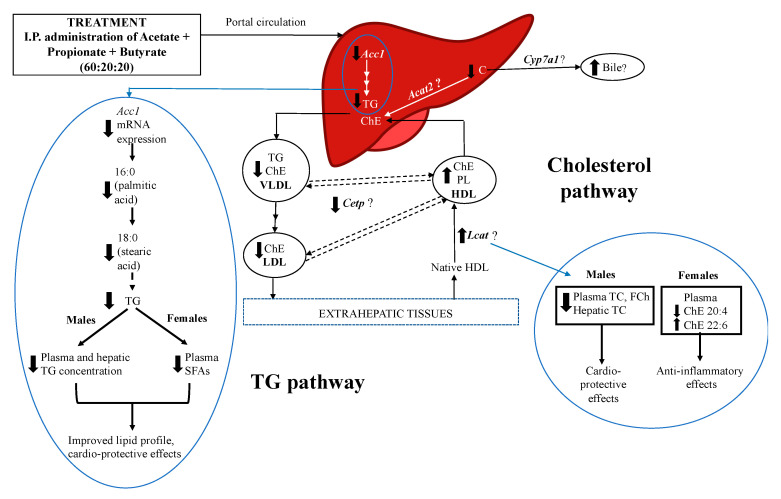
Proposed pathway by which SCFAs regulate lipid metabolism in a sex-specific manner in Long–Evans rats. Acetate, propionate, and butyrate, when administered intraperitoneally in a 60:20:20 ratio, enter portal circulation and reach the liver. Upon reaching the liver, SCFAs decrease the mRNA expression of *Acc1*, thereby decreasing plasma and hepatic TG levels in males, while altering TG species composed of saturated fatty acids (SFAs) in females; these alterations may elicit cardio-protective effects. SCFAs likely alter the lipid and fatty acid composition of lipoproteins, which may influence the activity of associated enzymes, such as Lcat and Cetp, and be responsible for reduced levels of plasma TC and FC in males. Although no changes were observed in *Cyp7a1* gene expression, it is possible that the bile acid production pathway is affected by SCFAs. SCFAs reduced the amount of arachidonic acid (*n*-6 PUFA), while increasing the amount of docosahexaenoic acid (*n*-3 PUFA) in ChE in only females, suggesting an anti-inflammatory effect. Overall, SCFAs may provide beneficial health effects to target metabolic disorders in both males and females via targeting the regulation of lipids and lipoprotein metabolism. *Acat*—acyl CoA:cholesterol acyl transferase, *Acc1*—acetyl CoA carboxylase, *Cetp*—cholesteryl ester transfer protein, ChE—cholesteryl esters, *Cyp7a1*—cholesterol 7a hydroxylase, FCh—free cholesterol, FFA—free fatty acids, HDL—high-density lipoprotein, I.P.—intraperitoneal, *Lcat*—lecithin cholesterol acyltransferase, LDL—low-density lipoprotein, PL—phospholipids, SCFAs—short-chain fatty acids, TC—total cholesterol, TG—triglycerides. Up and down arrows indicate an increase and decrease, respectively.

Our results indicate that SCFAs decreased plasma and hepatic TG levels in males, and the relative abundance of SFAs-composed TG species in females indicating beneficial effects of SCFAs on TG metabolism in both males and females, likely via independent pathways. In this study, the menstrual cycle was not synchronized for females. It has been suggested that metabolic traits such as thermogenesis, food intake, and locomotor activity may be different in females on specific days of their reproductive cycle due to the activation of the autonomous nervous system, rapid changes in neuronal firing, and secretion of certain neuropeptides involving sex hormone release [57]. These authors also suggested that the estrous cycle may be less relevant for other metabolic changes related to tissue function modification—lipid biology, insulin action, and adipose tissue storage. We did not observe any changes in food intake for any of our treatment groups; thus, it is unlikely that results were impacted by menstrual cycle. We have proposed a pathway for the sex-specific effects of SCFAs on cholesterol and TG metabolism, and lipid profile (Figure 6). Our findings suggest that upon reaching the liver, SCFAs decrease the mRNA expression of *Acc1* in males and females, thereby decreasing plasma and hepatic TC and TG levels. SCFAs also altered the fatty acid composition of ChE, PC, and TG species in females. Furthermore, it is possible that SCFAs directly or indirectly alter the activities of enzymes involved in lipid and lipoprotein metabolism, thereby affecting the composition of various lipid classes including phospholipids.

The proposed pathway may help identify the factors underlying sex-specific differences in TC and FC levels, and incorporation of PUFAs such as 20:4 and 22:6 by SCFAs.

## 5. Conclusions

Our findings show for the first time that a 60:20:20 molar ratio of acetate:propionate:butyrate, when administered via I.P., decreased the levels of plasma and hepatic TC, ChE, TG, and glucose in males, but not in females, compared to their respective controls. However, females showed more prominent effects of SCFAs on plasma and hepatic lipid fatty acyl composition. The decreased abundance of 20:4 ChE and TG molecular species composed of SFAs (18:0 and 16:0), and increased abundance of plasma PC containing PUFAs (20:4 and 22:6) indicates a possible role of SCFAs in modulating the inflammatory pathways in females. Positional differences in SFAs have been hypothesized to modulate the transport of *n-6* and *n-3* PUFAs [58]. Thus, SCFA-mediated changes in the fatty acyl composition of plasma TG may impact the fatty acid composition of complex lipids. It has been well established that the observed sex-specific effects on glucose and lipid metabolism are fundamentally due to the contribution of estrogen and progesterone in females and testosterone in males and/or XX/XY chromosome complement [59]. However, Sugiyama and Agellon [60] suggested that the differences in sex-specific lipid metabolism are not solely dependent on sex hormones and sex chromosomes, but on the metabolic fate of nutrient intake, which also contributes to this synergy. It is therefore possible that the SCFA mixture administrated via the I.P. route, upon reaching the liver, had different metabolism in males vs. females that led to differences in glucose, TC, and TG levels, along with alternations in fatty acyl incorporation. Furthermore, SCFA-treated males showed lower mRNA expression of *Acc1*, with a subsequent decrease in lipid levels, suggesting an SCFA-mediated suppression of *de-novo* lipogenesis. However, in females, SCFAs appear to have a role in inflammatory pathways by altering the *n*-6 and *n*-3 PUFA composition of lipids and lipoproteins. Thus, SCFAs may represent a suitable preventative/therapeutic strategy against metabolic disorders for both males and females; however, SCFAs may provide health benefits in both sexes via independent mechanisms.

## Figures and Tables

**Table 1:** Effect of short-chain fatty acids on body weight, organ weight (relative to body weight), glucose, and non-esterified fatty acid (NEFA) concentrations. Data were analyzed using two-way ANOVA followed by Tukey’s post hoc test. Values are presented as mean ± SD. Different superscripts indicate significant difference amongst groups; only the male treatment (MT) group showed decrease in glucose levels. *p* < 0.05 was considered significant (*n* = 6). FC—female control, FT—female treatment, MC—male control, SCFAs—short-chain fatty acids.

Parameter	MC	MT	FC	FT
Weight (g)				
Body weight (day 0)	271.3 ± 17.58 ^a^	273.2 ± 10.65 ^a^	198.78 ± 6.02 ^b^	198.26 ± 7.73 ^b^
Body weight (day 7)	300.33 ± 18.79 ^a^	301.83 ± 8.57 ^a^	214 ± 7.72 ^b^	203.66 ± 5.58 ^b^
Food intake	23.00 ± 1.75 ^a^	22.33 ± 1.87 ^a^	14.60 ± 1.07 ^b^	14.80 ± 0.88 ^b^
Liver	12.58 ± 1.24 ^a^	12.72 ± 1.24 ^a^	8.49 ± 0.45 ^b^	8.97 ± 0.44 ^b^
Abdominal fat	2.03 ± 0.56 ^ab^	2.34 ± 0.43 ^a^	1.74 ± 0.28 ^ab^	1.43 ± 0.26 ^b^
Concentration (mmol/L)				
Glucose	6.38 ^a^	4.76 ^b^	5.52 ^b^	6.01 ^ab^
NEFA	0.42	0.39	0.37	0.34

## Supplementary Materials

The following are available online at https://www.mdpi.com/2072-6643/13/3/892/s1, Figure S1: Intraperitoneal administration of SCFAs had no effect on hepatic ChE fatty acyl composition, Figure S2: Intraperitoneal administration of SCFAs mixture had no effect on TG metabolism in adipose tissue, Figure S3: Intraperitoneal administration of SCFAs showed no effect on liver TG fatty acyl composition, Figure S4: Intraperitoneal administration of SCFAs showed no effect on liver PC fatty acid composition in both, males and females, Figure S5: Intraperitoneal administration of SCFAs showed no effect on plasma LPC fatty acid composition in both, males and females but had significant effect on liver LPC in in females. Table S1: Primers for the gene of interest and housekeeping genes, Table S2: Parameters used for Orbitrap mass spectrometer.
