# Supplementary material for: Intraperitoneal Administration of Short-Chain Fatty Acids Improves Lipid Metabolism of Long–Evans Rats in a Sex-Specific Manner
Source: Nutrients. 2021 Mar 10;13(3):892. doi: 10.3390/nu13030892 (PMC8000072; doi:10.3390/nu13030892)
Supplement: Supplementary file 1 [file nutrients-13-00892-s001.pdf]

Supplementary Table 1 - Primers for the gene of interest ad housekeeping genes

Name of the gene	Base pairs	Gene sequence	Primer Efficiency (%)	Gene accession number
HMG CoA reductase (Hmgcr)	98	Forward ATT GCA CCG ACA AGA AAC CTG CTG<br><br>Reverse TTC TCT CAC CAC CTT GGC TGG AAT	119.72	NM_013134.2
Acetyl CoA carboxylase (*Acc1*)	101	Forward TGT AGA AAC CCGAAC CGT GG<br><br>Reverse CTG GAA ACC AAA CTT GC CG	115.03	NM_022193.1
Fatty acid synthase (*Fas*)	98	Forward GCT GCT ACA AAC AGG ACC AT<br><br>Reverse TCC ACT GAC TCT TCA CAG ACC A	110.45	NM_017332.1
Cholesterol 7α hydroxylase (*Cyp7a1*)	89	Forward ACG CAC CTC GCT ATT CTC<br><br>Reverse AGG CTG CTT TCA TTG CTT CA	107.83	NM_012942.2
Peroxisome proliferator activated receptor (*Pparg*)	89	Forward TGA TAT CGA CCA GCT GAA CC<br><br>Reverse TCA GCG GGA AGG ACT TTA TG	112.17	NM_013124.3
β-actin (*Actb*)	85	Forward AGC GTG GCT ACA GCT TCA CC<br><br>Reverse AAG TCT AGG GCA ACA TAG CAC AGC	114.13	NM_031144.3
Glyyceraldehyde-3-phosphate dehydrogenase (*Gapdh*)	99	Forward TGC CAC TCA GAA GAC TGT GG<br><br>Reverse TTC AGC TCT GGG ATG ACC TT	97.87	NM_017008.4

Supplementary Table 2: Parameters used for Orbitrap mass spectrometer

Parameter	
Sheath gas	40
Auxiliary gas	2
Ion spray voltage	3.2 kV
Capillary temperature	300 ^{o}C
S-lens RF	30 V
Mass range	200-2000 m/z
Resolution (full scan mode)	70,000 m/z
Resolution (top-20 data dependent MS/MS)	35,000 m/z
Collision energy	35 (arbitrary unit)
Injection time	35 minutes
Isolation window	1 m/z
Automatic gain control	1 e5 (w/ dynamic exclusion setting of 5.0s)
Target database	Q-Exactive
Precursor tolerance	5 ppm
Product tolerance	5ppm
Product ion threshold	5%
m-score threshold	2%
Quan m/z tolerance	±5 ppm
Quan RT range	1 minute
Adduct ions for positive ion mode	[M+H]+ and [M+NH4]+
Adduct ions for negative ion mode	[M-H]-, [M+HCOO]-, [M+CH3COO]- and [M-2H]2-

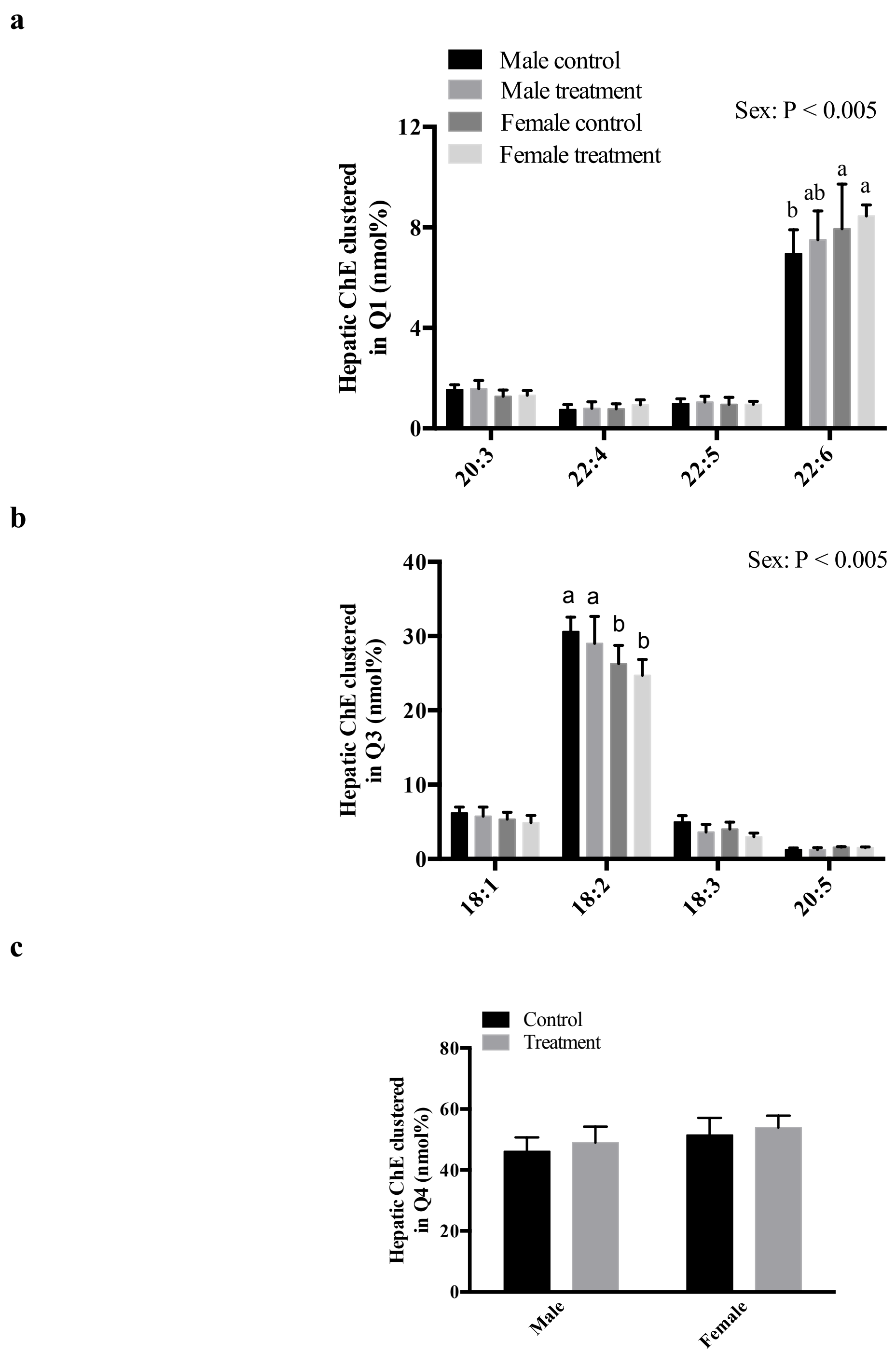

Supplementary Figure I – Intraperitoneal administration of SCFAs had no effect on hepatic ChE fatty acyl composition. TG molecular species clustered in; a) Q1 - quadrant 1, b) Q3 – quadrant 3 and c) Q4 – quadrant 4. Data were analyzed using multi-variant analysis and two-way ANOVA followed by Tukey's *post hoc* test. All data are expressed in mean ± SD. Different superscripts indicate significant difference amongst groups. $P < 0.05$ was considered significant (n=6). ChE – cholesteryl esters, SCFAs – short chain fatty acids.

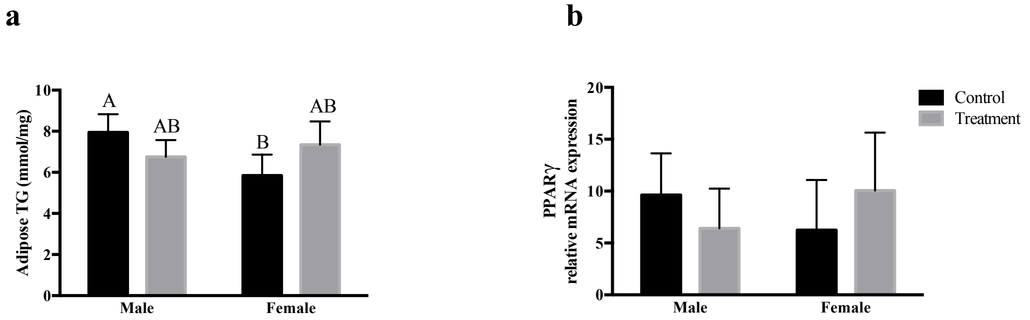

Supplementary Figure II – Intraperitoneal administration of SCFAs mixture had no effect on TG metabolism in adipose tissue. Effect of SCFAs on, a) adipose TG, b) the relative mRNA expression of *Ppag*. Data were analyzed using two-way ANOVA followed by Tukey's *post hoc* test. The mRNA expression data is presented as relative to *Gapdh*. All data are expressed as mean ± SD. Different superscripts indicate significant difference among groups. $P < 0.05$ was considered significant (n=6). *Gapdh* – glyceraldehyde 3-phosphate dehydrogenase, *Pparg* - peroxisome proliferator-activated receptor-γ, SCFAs – short chain fatty acids, TG – triglycerides.

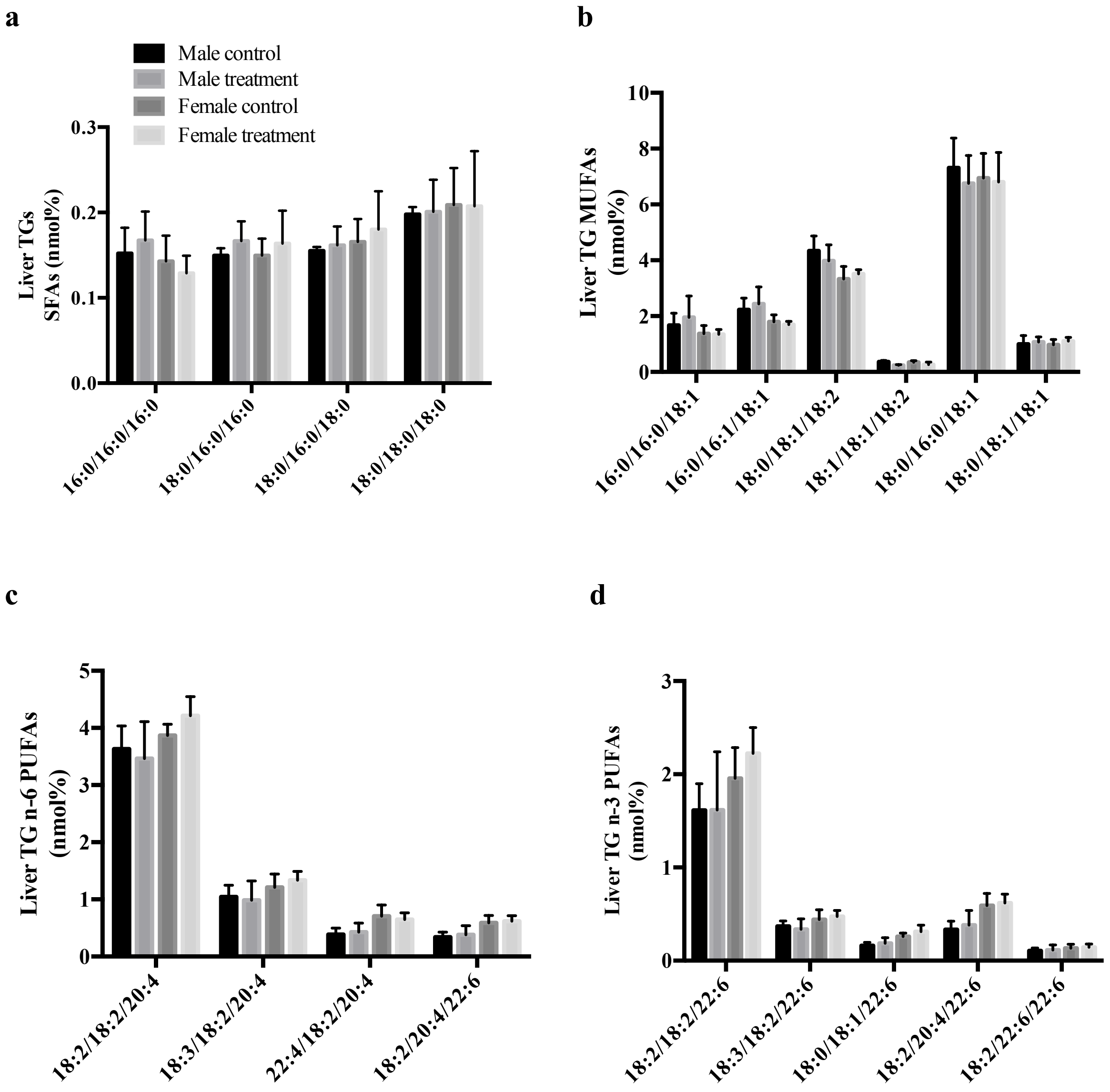

Supplementary Figure III – Intraperitoneal administration of SCFAs showed no effect on liver TG fatty acyl composition. TG molecular species composed of; a) SFAs, b) MUFAs, c) n-6 PUFAs and d) n-3 PUFAs. Data were analyzed using multi-variant analysis and two-way ANOVA followed by Tukey's *post-hoc* test. All data are expressed as mean ± SD. Different superscripts indicate significant difference amongst groups. $P < 0.05$ was considered significant (n=6). MUFAs – monounsaturated fatty acids, PUFAs – polyunsaturated fatty acids, SCFAs – short chain fatty acids, SFAs – saturated fatty acids, TG – triglycerides.

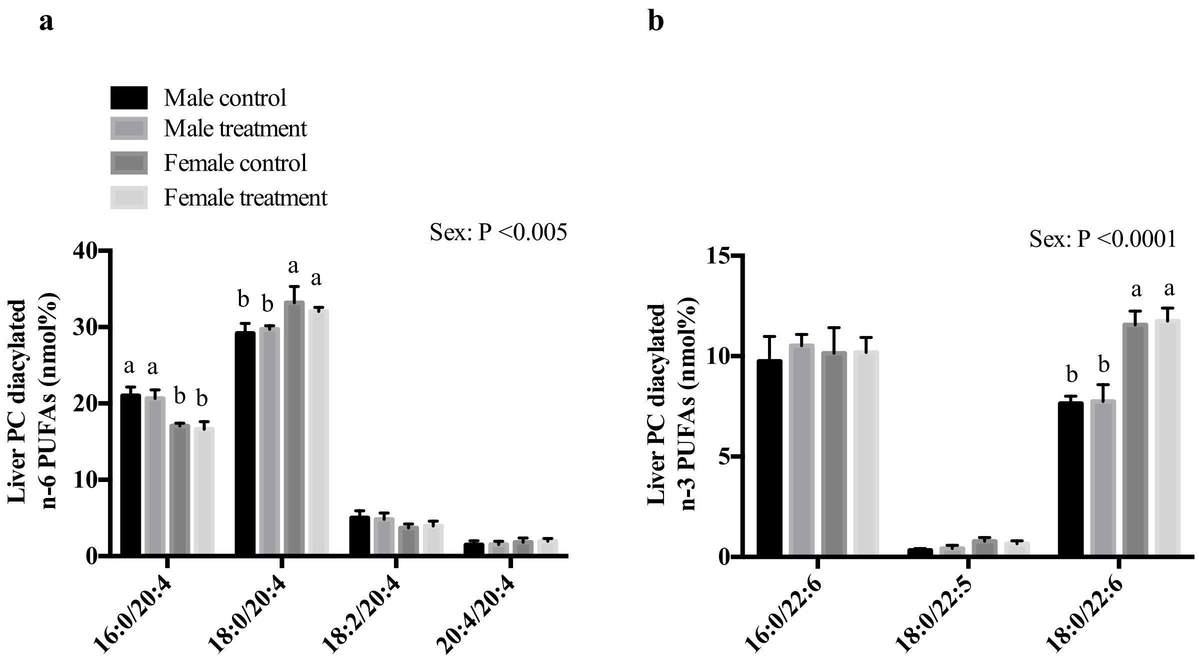

Supplementary Figure IV – Intraperitoneal administration of SCFAs showed no effect on liver PC fatty acid composition in both, males and females. PC species composed of; a) n-6 PUFAs, b) n-3 PUFAs. Data were analyzed using multivariate analysis and two-way ANOVA followed by Tukey's *post hoc* test. All data are expressed as mean ± SD. Different superscripts indicate significant difference amongst groups. $P < 0.05$ was considered significant (n=6). PC – phosphatidylcholine, PUFAs – polyunsaturated fatty acids, SCFAs – short chain fatty acids.

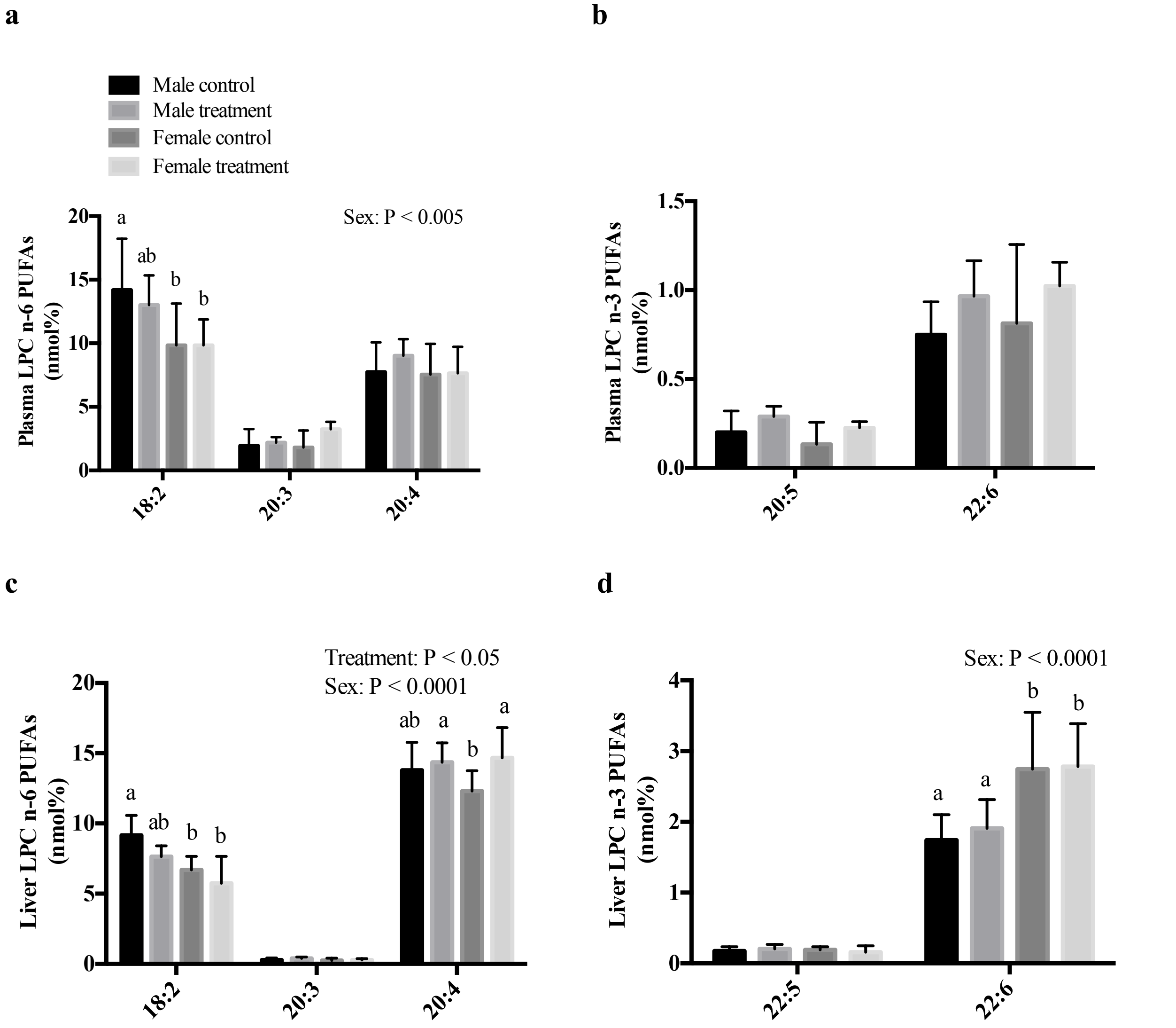

Supplementary Figure V – Intraperitoneal administration of SCFAs showed no effect on plasma LPC fatty acid composition in both, males and females but had significant effect on liver LPC in in females. LPC species composed of; a) n-6 PUFAs, b) n-3 PUFAs in plasma, c) n-6 PUFAs and d) n-3 PUFAs in liver. Data were analyzed using multivariate analysis and two-way ANOVA followed by Tukey's *post hoc* test. All data are expressed as mean ± SD. Different superscripts indicate significant difference amongst groups. $P < 0.05$ was considered significant (n=6). LPC – lyso phosphatidylcholine, PUFAs – polyunsaturated fatty acids, SCFAs – short chain fatty acids.
